# The complete chloroplast genome of desert spiny semi-shrub *Alhagi sparsifolia* (Fabaceae) from Central Asia

**DOI:** 10.1080/23802359.2020.1797558

**Published:** 2020-07-30

**Authors:** Ai-Hua Wang, Shuang-Wen Deng, Lei Duan, Hong-Feng Chen

**Affiliations:** a Key Laboratory of Plant Resources Conservation and Sustainable Utilization, South China Botanical Garden, Chinese Academy of Sciences, Guangzhou, China; b Key Laboratory of Environment Change and Resources Use in Beibu Gulf, Ministry of Education, Nanning Normal University, Nanning, PR China

**Keywords:** Chloroplast genome, Fabaceae, *Alhagi sparsifolia*, sand-resistant subshrub

## Abstract

*Alhagi sparsifolia* is a sand-resistant subshrub and food resource for camels in the desert and semi-desert areas of Central Asia. In China, this is the olny *Alhagi* species and it is restricted in the Northwestern region. Its complete chloroplast genome was sequenced using the Illumina Hiseq X-Ten platform. The genome lacks an inverted repeat (IR) region, containing 74 protein-coding genes, 30 *tRNAs* genes, and four *rRNAs*. The overall GC content is 43.6%. Based on the chloroplast genome sequence, a maximum-likelihood (ML) tree was constructed along with its 15 taxa, indicating that *A. sparsifolia* belong to the tribe Hedysareae, which nested in IRLC group of the subfamily Papilionoideae (Fabaceae).

*Alhagi* Gagnebin (Fabaceae), contain 3–5 species, naturally distribute in Mediterranean region, Western Asia, Caucasus, Central Asia, Northern South Asia, Himalayas, Mongolia, and Northwest China (Duan 2015). In China, Alhagi sparsifolia Schap. is the only species of this genus recorded in the Northwest (Xinjiang, Qinghai, Ningxia, Inner Mongolia, etc.) (Duan et al. 2015). As a perennial subshrub with deep strong roots, it can prevent wind and fix sand in desert and oasis (Jin 1994; Ma, Li, et al. 2018). Its leaves are food sources for camels, its seeds and chemical constituents are often used as medicine in locality (Ma, Shi, et al. 2018; Muratova et al. 2019). Few study focused on the genome of *A. sparsifolia* except Wu et al. (2015) made a transcriptomic analysis of its primary roots. A good knowledge in complete chloroplast genomic information of this species would contribute to the study of population genetics, diversity, medical use, and the establishment of efficient protection strategy.

Fresh leaves of the sample were collected from Turpan (Xinjiang, China) and preserved in silica gel. The voucher specimen ‘*L.Duan 2016021*’ was deposited in the South China Botanical Garden (IBSC). Total genomic DNA was extracted from silica-dried leaves referring to CTAB method (Doyle 1987). Then we fragmented them into about 500 bp in size. The genomic library was prepared and sequenced on the Illumina Hiseq X-Ten platform (Illumina Inc., San Diego, CA). The adapters of the raw data were removed by Trimmomatic (Bolger et al. 2014). SPAdes version 3.11 (Bankevich et al. 2012) was applied for the complete chloroplast (cp) genome *de novo* assembly. The cp genome was annotated by Dual Organellar GenoMe Annotator (DOGMA) (Wyman et al. 2004) and deposited in GenBank (accession number: MT571455).

About 1.33 Gb raw reads were obtained in total, with coverage of 1779× and 128,233 bp in length. The cp genome lacked inverted repeat (IR) region. The genome contained 74 protein-coding genes (CDS), 30 transfer *RNA* genes (tRNA), four ribosomal RNA genes (rRNA), within which 15 genes (*atpF*, *clpP*, *ndhA*, *petB*, p*etD*, *rpl2*, *rpl16*, *rpoC1*, *rps12*, *trnA-UGC*, *trnG-UCC*, *trnI-GAU*, *trnK-UUU*, *trnL-UAA*, and *trnV-UAC*) had one intron, one gene (*ycf3*) had two introns. Overall GC content of the whole genome was 43.6%.

To infer the phylogenetic relationship of this newly sequenced *A. sparsifolia* with related genera, 14 chloroplast genomes were downloaded from GenBank and were applied to construct the systematic tree. All accession numbers of these download genomes are shown in Figure 1. Geneious prime and Mauve plugin were used to adjust gene order and remove one copy of IR regions of chloroplast genomes. We aligned these 15 cp genomes using MAFFT version 7 (Katoh and Standley 2013) and generated a maximum-likelihood (ML) tree through the program IQ-TREE version1.4.2 (Nguyen et al. 2015). The result (Figure 1) showed that *Alhagi* has a close relationship with *Hedysarum*, and they undoubtedly belong to the tribe Hedysareae. Broadly speaking, the genus affiliate with the inverted repeat-lacking clade (IRLC) of the subfamily Papilionoideae, which is congruent with the previous study (Amirahmadi et al. 2014; Duan et al. 2015; Li et al. 2020).

**Figure 1.**
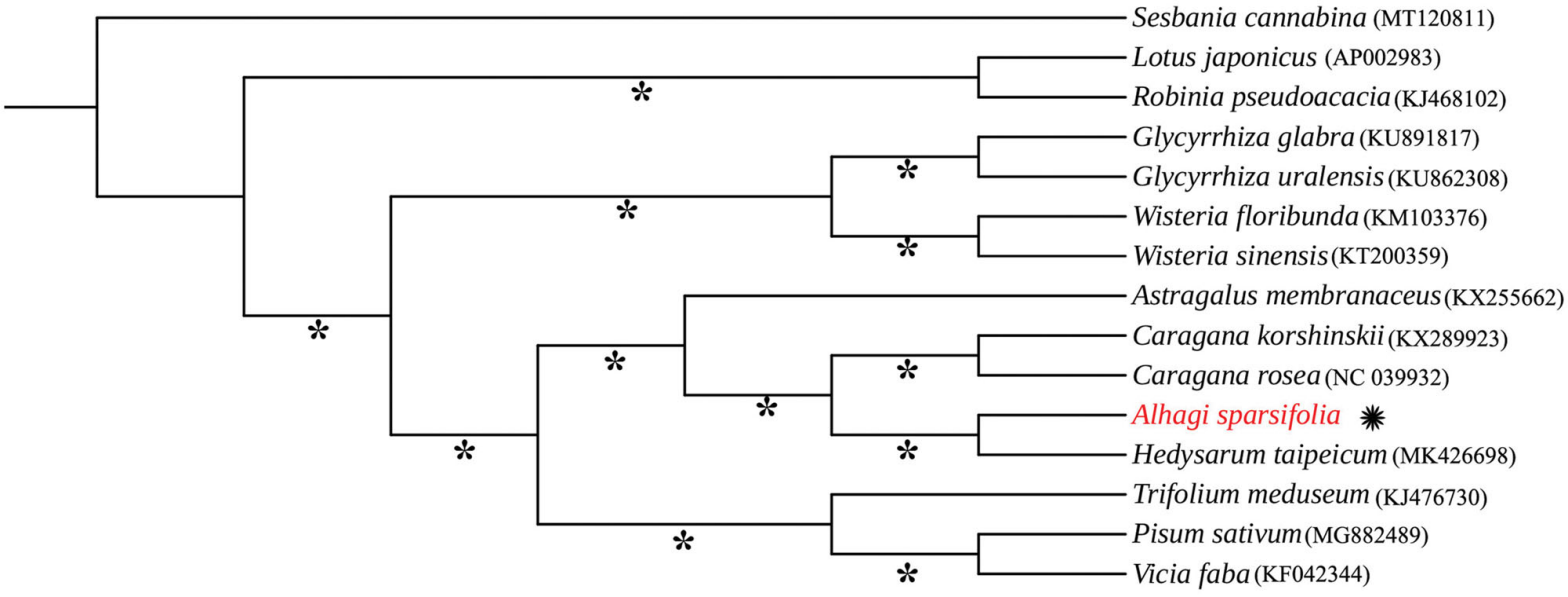
Maximum-likelihood phylogenetic tree inferred from 15 chloroplast genomes of Fabaceae. The position of *A. sparsifolia* is highlighted. The bootstrap values are shown above each node and the values of 100% are shown with asterisks.

## Data Availability

The data that support the findings of this study are openly available in GenBank at https://blast.ncbi.nlm.nih.gov/
